# Remote Ischemic Postconditioning Ameliorates the Mesenchymal Stem Cells Engraftment in Reperfused Myocardium

**DOI:** 10.1371/journal.pone.0146074

**Published:** 2016-01-13

**Authors:** Qin Jiang, Tao Yu, Keli Huang, Jing Lu, Hao Zhang, Shengshou Hu

**Affiliations:** 1 Department of Cardiac Surgery, Sichuan Provincial People’s Hospital, Affiliated Hospital of University of Electronic Science and Technology, Chengdu, China; 2 Department of Anesthesiology, Sichuan Provincial People’s Hospital, Affiliated Hospital of University of Electronic Science and Technology, Chengdu, China; 3 State Key Laboratory of Cardiovascular Disease, Fuwai Hospital, National Center for Cardiovascular Disease, Chinese Academy of Medical Sciences and Peking Union Medical College, Beijing, China; Indiana University School of Medicine, UNITED STATES

## Abstract

**Objectives:**

Remote Ischemic postconditioning (RIPoC) is a cardioprotective strategy for alleviating the reperfusion injury. We hypothesized that RIPoC or ischemic postconditioning (IPoC) could protect the engrafted mesenchymal stem cells (MSCs) in reperfusion myocardium.

**Methods:**

Female Sprague-Dawley rats were subject to 30 minutes of occlusion of left anterior descending (LAD). Ischemia reperfusion (IR) received reperfusion without interruption after ischemia. RIPoC received 3 cycles of 30 seconds reperfusion and re-occlusion on the limb at the onset of reperfusion. IPoC received 3 cycles of 30 seconds reperfusion and re-occlusion on the LAD at the same time. Male MSCs were intramyocardially administered after ischemia.

**Results:**

Compared with that in IR group, ischemic myocardium in RIPoC+IPoC group, RIPoC group and IPoC group were found to have higher anti-oxidative stress and mitochondrial function level, lower lipid peroxidation and inflammational injury level, higher level of stromal cell derived factor-1 alpha and vascular endothelium growth factor gene expression at 3 days later. By immunohistochemical examination and quantitative polymerase chain reaction, more engrafted MSCs, better cardiac function and less cardiac fibrosis in RIPoC+IPoC group, RIPoC group and IPoC group were detected at 3 weeks after delivery. There were no significant differences between RIPoC and RIPoC+IPoC group.

**Conclusions:**

Combination therapy using intramyocardial MSCs transplantation with RIPoC enhanced transplantation efficiency and cardiac function, and reduced cardiac fibrosis. These beneficial effects were mainly attributed to hospitable milieu for engrafted cells. IPoC could not render additional effect on MSCs engraftment elicited by RIPoC.

## Introduction

Reperfusion is the only way to salvage the ischemic myocardium but can paradoxically result in additional injury though timely restoration of coronary blood flow [1, 2].Ischemic conditioning is a well described adaptive response that markedly enhances the ability of the heart to withstand a prolonged ischemia reperfusion (IR) insult and provides therapeutic paradigms for cardioprotection[3]. In the majority of phase II studies published to date, ischemic postconditioning evoked a≈35% reduction of infarct size in ST-segment-elevation myocardial infarction patients[4]. Remote ischemic postconditioning (RIPoC) which administered with brief, non-lethal episodes of ischemia in another organ was also demonstrated to be cardioprotective against myocardial ischemia [4,5]. However, combined remote ischemic perconditioning and local ischemic conditioning strategy in ST-segment elevation myocardial infarction study did not lead to further decrease in infarct size [6].

Intramyocardial mesenchymal stem cells (MSCs) transplantation showed promising efficacy on left ventricular function recovery after myocardial ischemia [7]. But its engraftment and retention were hampered by harsh milieu with oxidative stress and inflammatory reaction resulting from IR [8]. The endangered MSCs could furthermore attenuate the therapeutic effect for IR insult.

In the previous study, we concluded that RIPoC could recruit intravenously infused MSCs into ischemic heart by SDF-1α/CXCR4 axis [9]. The aim of this study was to investigate whether RIPoC could enhance intramyocardial MSCs engraftment and therapeutic efficacy by ameliorating the deteriorated milieu. Meanwhile, it also explored whether combination of RIPoC and IPoC could provide an additional effect on MSCs intramyocardial engraftment.

To clarify the effect of oxidative stress injury on MSCs retention in myocardium, we quantified the number of intramyocardially delivered MSCs with ROS scavenger N-acetylcyseine in the presence of ischemic reperfusion. The ATP level, mitochondrial function, and inflammation were also detected. In addition, we examined the expression of factors including SDF-1α and VEGF, which potentially involved in the homing and retention of stem cells in the myocardium.

## Materials and Methods

### Animals and ethic declaration

Animals were purchased from Vital River Company (Beijing, China). All the animal experiments were approved by the ethic committee of Sichuan provincial people’s hospital and conducted in accordance with the guidance for care and use of laboratory animals published by the UK Home Office guidelines on the Animals (Scientific Procedures) Act 1986 (The Stationary Office London, UK) and the US National Institutes of Health (NIH Publication No. 85–23, revised 2010).

### Mesenchymal stem cells preparation

All the MSCs for engraftment experiments were harvested from 60 gram male *Sprague-Dawley(SD) rats*. In brief, the animals were executed by dislocating the neck. Bone marrow cavities of femur and tibia were flushed with complete culture media under sterile conditions. The adhesive MSCs were cultured to the third generation while the non-adhesive cells were removed by changing fresh media. To trace the injected MSCs, the thymidine analogue 5-bromo-29 deoxyuridine (BrdU, Sigma-Aldrich, St. Louis, MO) was dissolved and added to culture medium overnight at a final concentration of 10 μmol/L [10]. The amounts of MSCs were numerated with cell counting instrument (Countess, Invitrogen, Carlsbad, CA) and the viability of the injected MSCs was tested with typan blue (Invitrogen, Carlsbad, CA).

### Acute myocardial ischemia-reperfusion injury model

Female *SD rats* (250-300g) were used and anesthetized by intraperitoneal injection of 10%chlora hydrate (0.35 ml/100g body weight). Different concentration of isoflurane was inhaled through an oxygen mask depending on the surgical stage (2% during painful stimuli, and 1% during latent periods). The animals were orally intubated with a 23-gauge vinyl catheter and ventilated via Harvard Model 683 Small Animal Ventilator(Harvard Biosicence Company, Holliston, MA) with a tidal volume of 1.0 ml per 100 gram body weight and a breath rate of 70 breaths per minute. A left thoracotomy was performed in the fourth left intercostals space. Left anterior descending artery (LAD) 2mm distant from tip of the normally positioned left auricle was tied with reversible knot by a 6–0 polyester suture attached to a small curved needle. Successful occlusion of LAD was ascertained by appearance of myocardial apex pallor and undermined beating. About ninety percent of animals survived and were randomized into the separate groups to receive different treatments after 30 min ischemia. All the animals were anesthetized as mentioned above and euthanized by intravenous injection of 1ml potassium chloride (with a concentration of 4 mmol/L) via the femoral vein for harvesting the heart.

### Experimental design

**Fig 1:**
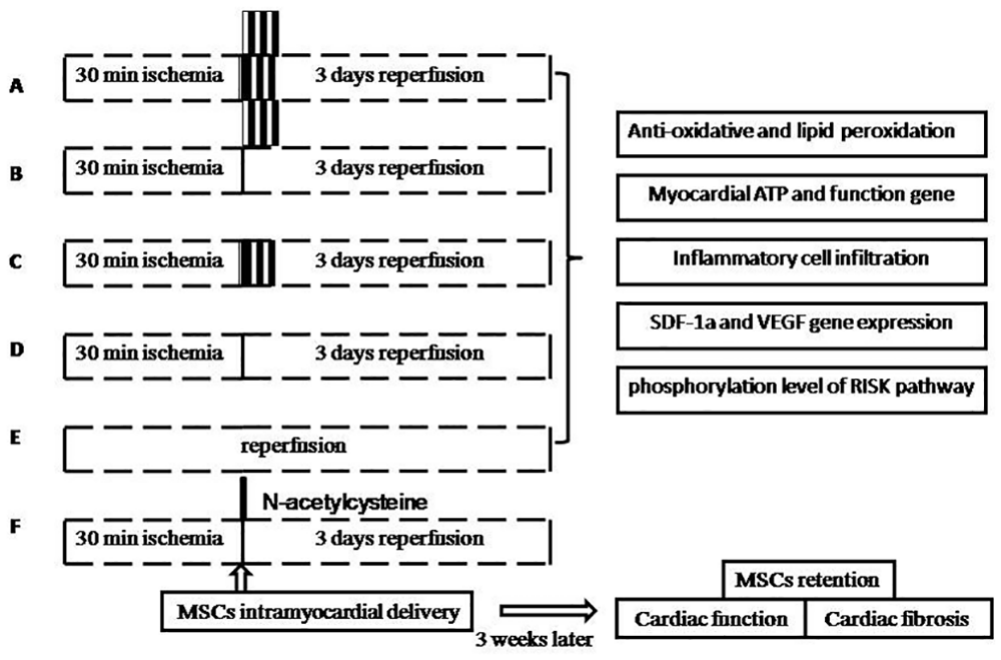
Experiment protocol. Group A: RIPoC+IPoC group, received the combined algorithms of RIPoC and IPoC; Group B: RIPoC group, which received 3 cycles of 30 seconds reperfusion and re-occlusion on the limb at the onset of reperfusion; Group C: IPoC group, received 3 cycles of 30 seconds reperfusion and re-occlusion on the LAD; Group D: IR group, received reperfusion without interruption after ischemia; Group E: sham group, received only open chest; Group F: NAC+IR group, ROS scavenger N-acetylcyseine was administered in IR group. Group A-E underwent ischemic heart detections including on anti-oxidative stress index, mitochondrial function, inflammation reaction, stem cell survival and homing. Group A-F underwent MSCs intramyocardial transplantation and cardiac function tests. MSCs: mesenchymal stem cells. SDF-1α: stromal cell derived factor-1 alpha. VEGF: vascular endothelium growth factor. RISK: reperfusion injury survival kinase. NAC: N-acetylcyseine.

Animals subject to ischemia received different types of reperfusion as illustrated in Fig 1. RIPoC group received 3 cycles of 30 seconds ischemia and reperfusion on the limb with tourniquet at the onset of reperfusion. IPoC group received 3 cycles of 30 seconds reperfusion and reocclusion on the LAD at the onset of reperfusion. RIPoC + IPoC group received the combined algorithms of RIPoC and IPoC.IRgroup (no intervention in the reperfusion period). sham group(only open chest surgery). The hearts from each group were harvested for all the basic experiments 3 days later (n = 4). In the separate experiment, another same five groups of animals received intramyocardial engraftment of MSCs (n = 16). To clarify the effect of oxidative stress injury on MSCs retention in myocardium, we quantified the number of intramyocardially delivered MSCs with ROS scavenger N-acetylcyseine in the model of IR. Injected cell was suspended into PBS solution (2×10^7^/ml).Cell suspension (100ul) was injected into three points around the borderline of myocardial ischemia with a 30 degree curved insulin syringe needle. After transplantation, all the animals received thoracic closure and were cultured in animal facilities for 3 weeks.

### Anti-oxidative stress and lipid peroxidation level

To assess the effect of different reperfusion strategies after myocardium ischemia, the levels of anti-oxidative stress and lipid peroxidation were compared among the five groups with their presumptive indicators, respectively (n = 4). The borderline zone of ischemic myocardium was harvested and homogenized. The enzymatic activities of superoxide dismutase (SOD, expressed as U/mg protein) and malondialdahyde (MDA, expressed as umol/g protein) were measured spectrophotometrically at the recommend wave length according to the manufacturer’s instructions (Nanjing Jiancheng Bioengineering Institute, Nanjing, China) as previously [11].Moreover, relative mRNA levels of the genes involved in anti-oxidative defense(SOD1, SOD2) were calculated as before [12]. The sequence of the primer sets is listed in Table 1. Quantitative analysis of gene expression was done using the GAPDH gene as the inner control.

**Table 1:** Primer sequences used for quantitative real-time polymerase chain reaction (RT-PCR) amplification.

Primers	Forward primer 5ˊ→3ˊ	Reverse primer 5ˊ→3ˊ
SOD1	AGATGACTTGGGCAAAGGTG	CAATCCCAATCACACCACAA
SOD2	CTGGACAAACCTGAGCCCTA	GAACCTTGGACTCCCACAGA
Cycs	CCAAATCTCCACGGTCTGTTC	ATCAGGGTATCCTCTCCCCAG
Ndufa8	GGAGCTGCCAACTCTGGAAG	CCAGCGGCACAGCATAAAC
Cox7a1	GCTCTGGTCCGGTCTTTTAGC	GTACTGGGAGGTCATTGTCGG
TFAM	ATTCCGAAGTGTTTTTCCAGCA	TCTGAAAGTTTTGCATCTGGGT
SDF-1α	TGAGAGCCATGTCGCCAGA	GGATCCACTTTAATTTCGGGTCAA
VEGFA	GTCCTCACTTGGATCCCGACA	CCTGGCAGGCAAACAGACTTC
GAPDH	GGCACAGTCAAGGCTGAGAATG	ATGGTGGTGAAGACGCCAGTA

### Measurement of ATP

The level of myocardial ATP was measured by using a bioluminescence method as previously described [13]. The myocardium samples were kept in the ATP assay buffer, and homogenized with 50 mmol/L Tris-acetate buffer containing 2 mmol/L EDTA (pH 7.75), 1% NP-40, 150 mmol/L NaCl, and 0.1% SDS. The homogenized tissue was centrifugated at 12,000 × g for 30 min to pellet insoluble materials. Then, the supernatant was added to a 96-well plate for ATP assessment. According to the manufacturer's instructions, myocardial ATP levels were assayed with ATP assay system (Nanjing Jiancheng Bioengineering Institute, Nanjing, China).

### Mitochondrial function-related genes

The mRNA expression levels of mitochondrial function-related genes *Cycs*, *Ndufa8*, *Cox7a1* and *TFAM* in separate groups were determined by real-time PCR as before[13]. The primers of these genes are also listed in Table 1.

### Myeloperoxidase assay

Myeloperoxidase(MPO), an enzyme present in leukocytes, was determined in boundary area around infarction with MPO kits as an index of tissue inflammation cell infiltration (Nanjing Jiancheng Bioengineering Institute, Nanjing, China)as previously described[14].

### The protein phosphorylation level of reperfusion injury salvage kinase(RISK) pathway

Protein was isolated from homogenized heart tissue (n = 4) with nondenaturing lysis buffer added with proteinase inhabitor reagent and the concentrations were calculated by BCA assay(Applygen Technologies Inc., Beijing, China). To determine the phosphorylated level ofAkt(serine[Ser] 473) and Erk1/2 in addition to the total level of the proteins(Cell SignalingTechnology, Beverly, MA), 40 μg homogenized protein was separated on a Bis-Tris Gel and transferred to a nitrocellulose mini membrane (Invitrogen, Carlsbad, CA). Membranes were then probed with a rabbit polyclonal antibody against rat Akt(serine[Ser] 473) and Erk1/2(Cell Signaling Technology, Beverly, MA). The bound primary antibodies were detected with horseradishperoxidase-conjugated secondary antibodies and enhanced chemi- luminescencedetection reagents (Applygen Technologies Inc., Beijing, China). The reported densitometry values were analyzed by Totalab v1.10 software (Totalab Ltd., Newcastle, U.K.) and normalized to total Akt and Erk1/2.

### Gene expression of stromal cell derived factor-1 alpha (*SDF-1α*) and vascularendothelium growth factor (*VEGF*)

To examine the gene expression of *SDF-1α* and *VEGF* in ischemic myocardium (n = 4), the total RNA were prepared by homogenizing tissue with TRIzol Reagent (Invitrogen, Carlsbad, CA). For reverse-transcription polymerase chain reaction, 1ug of total RNA was added to the reaction system according to manufacturer’s guideline (Promega, Madison, WI).The resulting cDNA was used as template in PCR with cDNA specific primers spanning at least one intron. The primers are listed in Table 1. For the RT reaction, the reaction condition was denaturation for 10 minutes at 70°C followed by 15 minutes at 42°C and 5 minutes at 95°C.For quantitative real-time PCR, 4 uL cDNA diluted 20-fold was used with Master Mix SYBR Green One (Applied Biosystems, Carlsbad, CA) and normalized to *GAPDH*.

### Immnohistochemical analysis of MSCs retention

Slides for immnohistochemical examination were prepared from formalin-fixed and paraffin-embedded hearts. Mouse monoclonal anti-BrdU antibody (Sigma-Aldrich, St. Louis, MO) was used to detect the labeled cells (n = 4). The cells with brown-stained nuclei were directly counted under a microscope (Olympus BX61, Tokyo, Japan) at 400-foldmagnification in ten positive fields from three distinct slides of each heart.

### Real-time polymerase chain reaction analysis of MSC retention

For absolute quantification of engrafted MSCs, the hearts were harvested at 3 weeks later (n = 8). The genomic DNA was isolated from the whole heart, the primer of *SRY* gene for detecting Y chromosome was as followed: forward, *5´-* CATCGAAGGGTTAAAGTGCCA*-3´*; and reverse, *5´-*ATAGTGTGTAGGTTGTTGTCC*-3´*. The real-time PCR conditions were an initial denaturation step of 10 min at 95°C, followed by 40 cycles of 95°C for 15 seconds and60°C for 1min on the ABI PRISM 7300 sequence detection system (Applied Biosystems, Carlsbad, CA). A dissociation curve was generated for all reactions, and one single crest verified the presence of primer specific amplification.

### Cardiac function

A specialized sonographer who was blinded to the experimental design utilized echocardiography (UCG) before and 3 weeks after cell transplantation to record cardiac function(n = 8). The left ventricular end-diastolic diameters (LVEDD) and left ventricular end-systolic diameters (LVESD) were measured from M-mode recordings. Left ventricular fractional shortening (LVFS) was calculated as: (LVEDD−LVESD) ×100/LVEDD [9].The results were the average of six selected cardiac cycles from two independent scans. Dimensions were measured between the anterior wall and the posterior wall in the short-axis view with papillary muscles as the reference point.

### Cardiac fibrosis

To evaluate the effect of MSCs on the myocardial remodeling, hematoxylin-eosin and masson staining (Sigma-Aldrich, St. Louis, MO) were done to measure the fibrosis area of LV 3 weeks after delivery. With the image analysis software Image-Pro Plus, the area of fibrosis was calculated as the area stained blue divided by the total area of the LV wall. The measurement was done in at least 3 independent sections of each heart (n = 4).

### Data and statistical analysis

All data were presented as mean ± SD. Statistical analyses were performed by using SPSS17.0. Statistical significance was evaluated with 2-tailed independent Student’s *t* test between2 groups and one-way analysis of variance (ANOVA) for multi-group comparisons. Differences were considered as statistically significant when p<0.05.

## Results

### MSCs preparation

MSCs formed patchy colonies with spindle-shaped fibroblast-like appearance in culture plates. The surface phenotype and the potential to differentiate towards multi-lineage were identified as before [10]. The efficiency of MSCs labeling with BrdU was nearly 100%. The cell number before delivery was calculated with COUNTESS instrument and the viability was 100% by trypan blue exclusion test.

### Experimental model mortality

On the basis of the experimental protocol, one hundred and twenty-two rats were used in this study. The death mainly happened during cardiac ischemia and MSCs injection period. There was not significantly different among six groups (9%, 2/22, 4.8%, 1/21, 9%, 2/22, 4.8%, 1/21, 0%, 0/20, 0%, 0/16, respectively, *p*> 0.05).

### The superoxide dismutase (SOD) and malondialdahyde (MDA) level

**Fig 2:**
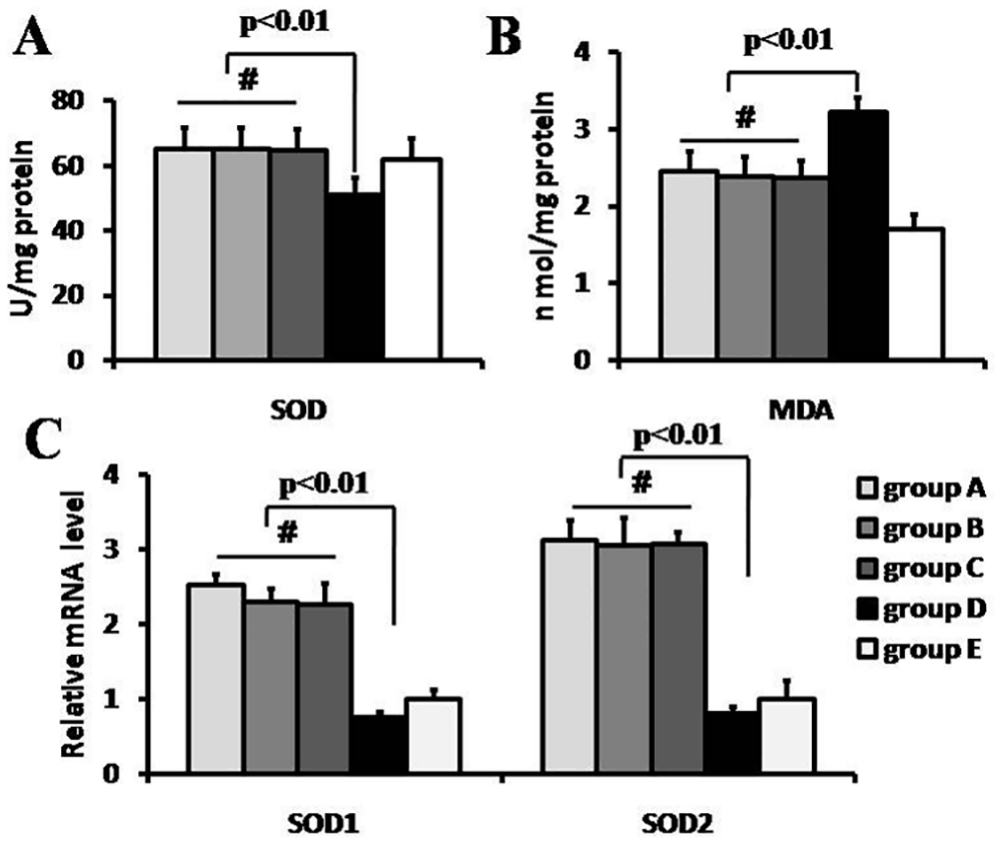
Antioxidative stress reaction and gene expression. The level of anti-oxidative stress(A) and lipid peroxidation(B) at 3 days after myocardium reperfusion (n = 4). RIPoC+IPoC, RIPoC and IPoC procedure increased the levels of anti-oxidative stress and decreased the lipid peroxidation reaction in reperfusion myocardium (p<0.01).The SOD1 and SOD2 gene expression level at the borderline of ischemic myocardium were detected(C). The gene expression of SOD1 and SOD2 were enhanced in RIPoC+IPoC, RIPoC and IPoC group compared with that in IR group (p<0.01). Group A: RIPoC+IPoC group, received the combined algorithms of RIPoC and IPoC; Group B: RIPoC group, which received 3 cycles of 30 seconds reperfusion and re-occlusion on the limb at the onset of reperfusion; Group C: IPoC group, received 3 cycles of 30 seconds reperfusion and re-occlusion on the LAD; Group D: IR group, received reperfusion without interruption after ischemia; Group E: sham group, received only open chest.

In the case of SOD concentration, there was no statistical difference among RIPoC + IPoC group, RIPoC group, or IPoC group (65.00±6.48, 65.25±6.24 vs. 64.50±6.55U/mg protein, p>0.05), but a significantly lower value in IR group (50.75±5.50U/mg protein) compared with that in RIPoC + IPoC group, RIPoC group and IPoC group (p<0.01, Fig 2A). On the contrary, the MDA level was remarkably lower in RIPoC + IPoC group, RIPoC group and IPoC group than that in IR group(2.44±0.26, 2.38±0.26 and 2.36±0.22 vs. 3.21±0.19 umol/g protein, respectively, p<0.01). No statistical difference was found in the concentration of MDA among in RIPoC + IPoC group, RIPoC group and IPoC group (p>0.05) (Fig 2B). The gene expressions of SOD1 and SOD2 were both increased in RIPoC + IPoC group, RIPoC group and IPoC group than that in IR group, respectively (p<0.01, Fig 2C).

### Mitochondrial function

**Fig 3:**
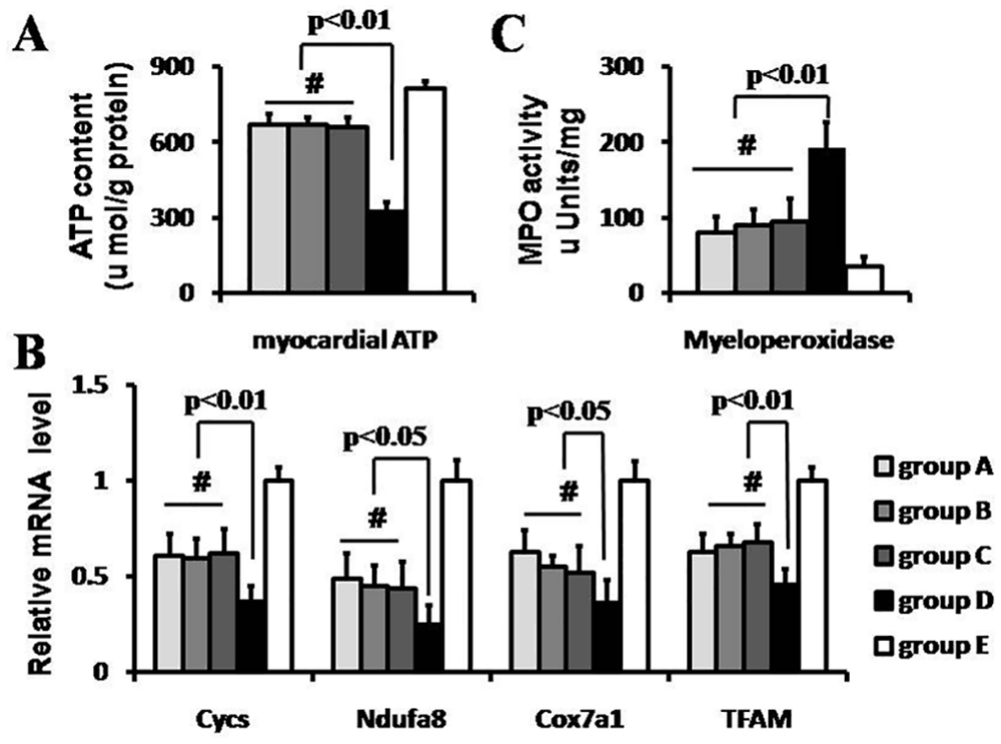
Mitochondrial function and inflammation reaction. The myocardial ATP level (A) and mitochondrial function gene expression levels (B) were also detected. The myocardial ATP level was higher in RIPoC+IPoC, RIPoC and IPoC group than that in IR group (p<0.01). The mRNA expression levels of *Cycs*, *Ndufa8*, *Cox7a1* and *TFAM* were significantly regulated up in RIPoC+IPoC, RIPoC and IPoC group than that in IR group. The myeloperoxidase(MPO) assay, which indexed inflammation reaction, also determined(C). The MPO level was lower in RIPoC+IPoC, RIPoC and IPoC group than that in IR group (p<0.01). Group A: RIPoC+IPoC group, received the combined algorithms of RIPoC and IPoC; Group B: RIPoC group, which received 3 cycles of 30 seconds reperfusion and re-occlusion on the limb at the onset of reperfusion; Group C: IPoC group, received 3 cycles of 30 seconds reperfusion and re-occlusion on the LAD; Group D: IR group, received reperfusion without interruption after ischemia; Group E: sham group, received only open chest.

In line with the myocardium subject to ischemia reperfusion injury, the myocardial ATP level in IR group was significantly lower than that in sham group (322.5±40.3 vs. 812.5±29.9umol/g prot, p<0.01, Fig 3A). And compared with that in IR group, the ATP content in RIPoC+IPoC group, RIPoC group and IPoC group increased by 104.7%, 107.8% and107.8%, respectively (p<0.01). The transcriptional levels of mitochondrial function-related genes were assessed next. Compared with that in sham group, the mRNA levels of *Cycs*, *Ndufa8*, *Cox7a1* and *TFAM* were decreased in IR, RIPoC, IPoC and RIPoC+IPoC groups, respectively (p<0.05, Fig 3B). Besides, compared with that in IR group, the levels of the *Cycs* mRNA in RIPoC+IPoC, RIPoC and IPoC group increased by 67.6%, 64.9% and60.8%, respectively (p<0.01); the levels of the *Ndufa8* mRNA in RIPoC+IPoC, RIPoC and IPoC group increased by 76.0%, 96.0% and80.0%, respectively (p<0.05);the levels of the *Cox7a1* mRNA in RIPoC+IPoC, RIPoC and IPoC group increased by 44.4%, 75.0% and 52.8%, respectively(p<0.05); the levels of the *TFAM* mRNA in RIPoC+IPoC, RIPoC and IPoC group increased by 47.8%, 37.0% and43.5%, respectively (p<0.01).

### Inflammation reaction

MPO activity in ischemic tissue was significantly increased in RIPoC+IPoC group (80.0±21.6 μU/mg), RIPoC group (90.0±21.6 μU/mg) and IPoC group (95.0±31.1 μU/mg) compared to that in IR group (192.0±34.0 μU/mg,p<0.01) (Fig 3C). But MPO activity in RIPoC+IPoC group was not significantly less than that in RIPoC group or IPoC group.

### The Phosphorylation level of Akt and Erk1/2

**Fig 4:**
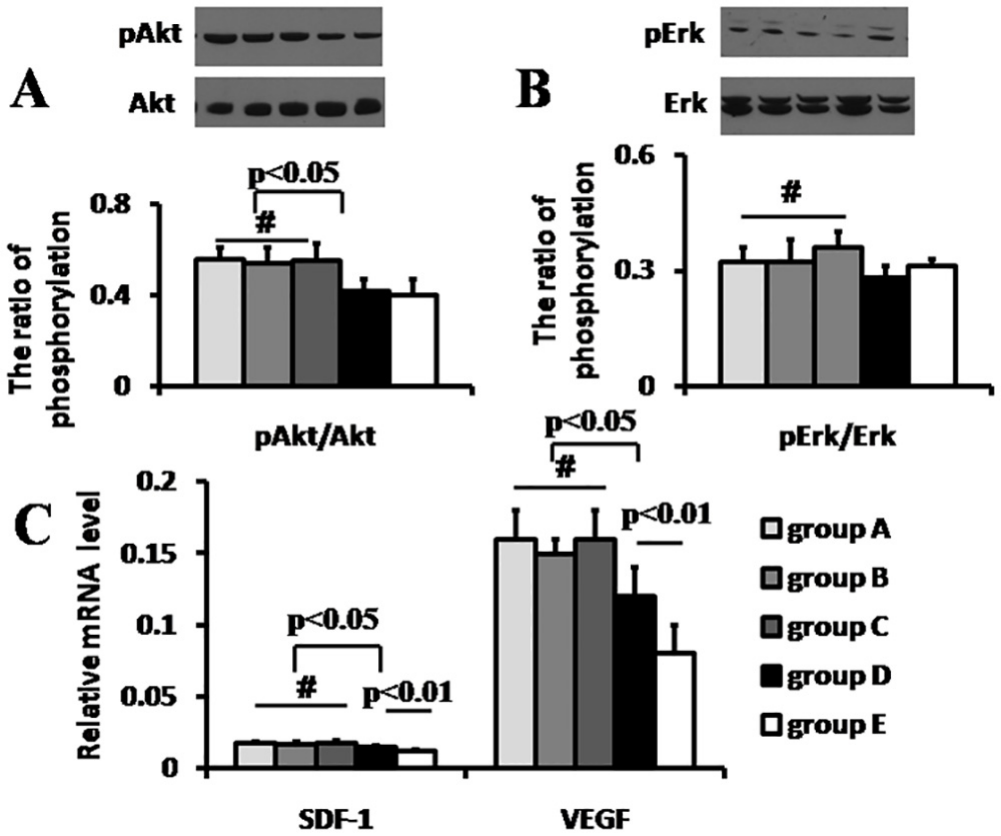
RISK pathway protein phosphorylation and gene expression of stem cell retention and survival. The protein phosphorylation level in RISK pathway. The pAkt(A) but not Erk(B) phosphorylation level was higher in RIPoC+IPoC, RIPoC and IPoC group than that in IR group (p<0.05). The gene expression of stem cell retention (stromal cell derived factor-1 alpha, SDF-1α, p<0.05) and survival (vascular endothelium growth factor, VEGF, p<0.05) was up-regulated in RIPoC+IPoC, RIPoC and IPoC group than that in IR group(C). Group A: RIPoC+IPoC group, received the combined algorithms of RIPoC and IPoC; Group B: RIPoC group, which received 3 cycles of 30 seconds reperfusion and re-occlusion on the limb at the onset of reperfusion; Group C: IPoC group, received 3cycles of 30 seconds reperfusion and re-occlusion on the LAD; Group D: IR group, received reperfusion without interruption after ischemia; Group E: sham group, received only open chest.

The western blotting analysis showed that the total Akt and Erk1/2 expression level was comparable in the five groups (p>0.05). It also revealed that the phosphorylated level of Akt protein on the site of serine 473 was statistically higher in RIPoC + IPoC group, RIPoC group and IPoC group than that in IR group, respectively (0.56±0.05, 0.54±0.07and 0.55±0.08 vs. 0.42±0.05, p<0.05)(Fig 4A). No significant difference in the phosphorylated Erk1/2 among RIPoC + IPoC group, RIPoC group, IPoC group, or IR group (0.32±0.04, 0.32±0.06 and 0.36±0.04 vs. 0.28±0.03, p>0.05) (Fig 4B).

### The gene expression level of *SDF-1α* and *VEGF*

In terms of gene expression of *SDF-1α* normalized by control genes, RIPoC +IPoC group, RIPoC group and IPoC group had higher value than that of IR group (1.74±0.09%, 1.73±0.15% and 1.78±0.15% vs. 1.51±0.10%, p<0.05). Similarly, RIPoC + IPoC, RIPoC group and IPoC group had a remarkable increase in the mRNA level of *VEGF* than that of IR group (16±2.0%, 15±1.0% and 16±2.0% vs. 12±2.0%, p<0.05) (Fig 4C). The expression level of *SDF-1α* and *VEGF* among RIPoC + IPoC group, RIPoC group and IPoC group was no remarkable differences, respectively(p>0.05).

### The retention of the engrafted MSCs

**Fig 5:**
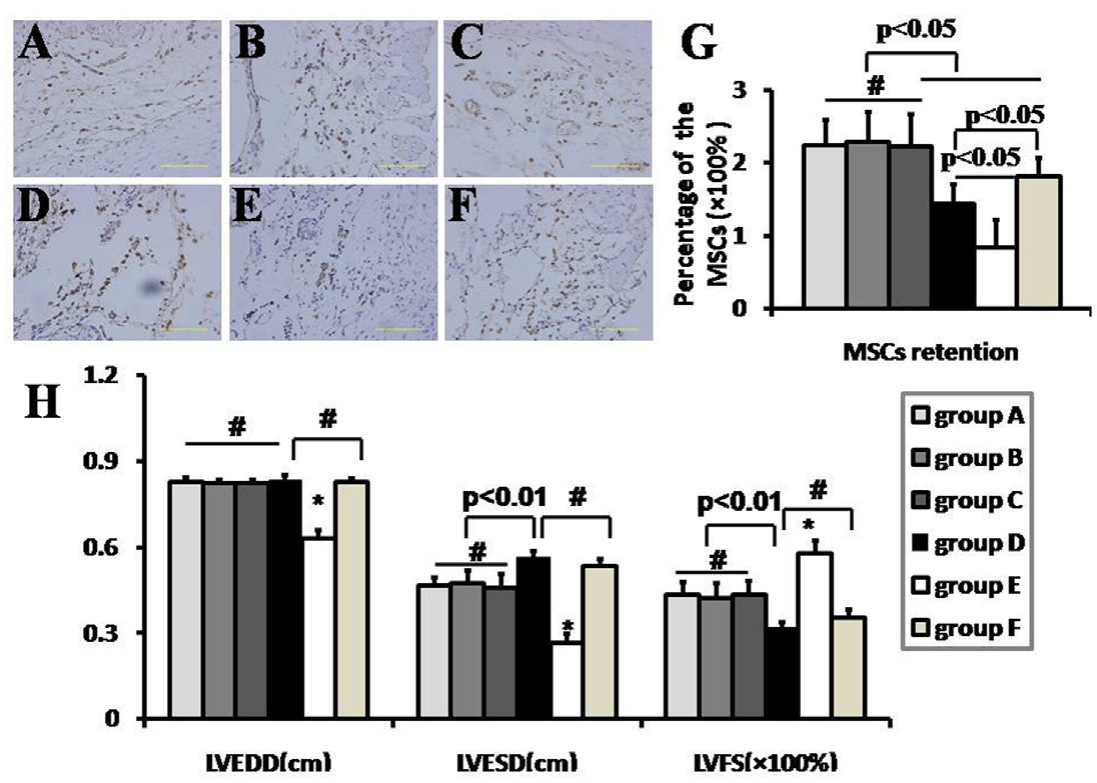
MSCs retention in myocardium and cardiac function detection. Cell retention by anti-brdu antibody immnohistochemical examination(A-F) and PCR quantification for SRY gene(G), and cardiac function detection(H) at 3 weeks after mesenchymal stem cells(MSCs) intramyocardial delivery (n = 8). The MSCs retention was least in sham group (p<0.05). More MSCs was calculated in RIPoC+IPoC, RIPoC and IPoC group than that in IR group (p<0.05). More retention after NAC administration compared with that in IR group, but lower retention than that in RIPoC+IPoC, RIPoC and IPoC group. LVESD was smaller in RIPoC+IPoC, RIPoC and IPoC group than that in IR group (p<0.01). LVFS was higher in RIPoC+IPoC, RIPoC and IPoC group than that in IR group (p<0.01). However, NAC administration didn’t significantly enhance cardiac function. The cells with brown-stained nuclei indicated the Brdu-labeled positive MSCs (Fig 5A). Bar indicated 100 um. The magnification was ×400 (n = 4). # indicated p>0.05, * indicated p<0.05. Group A: RIPoC+IPoC group, received the combined algorithms of RIPoC and IPoC; Group B: RIPoC group, which received 3 cycles of 30 seconds reperfusion and re-occlusion on the limb at the onset of reperfusion; Group C: IPoC group, received 3 cycles of 30 seconds reperfusion and re-occlusion on the LAD; Group D: IR group, received reperfusion without interruption after ischemia; Group E: sham group, received only open chest; Group F: NAC+IR group, ROS scavenger N-acetylcyseine was administered in IR group.

The number of Brdu-labeled positive cells in sham group was significantly lower than that in IR group (p<0.05). No remarkable difference in the amount of brown-staining cells between RIPoC + IPoC group, RIPoC group and IPoC group (p>0.05, Fig 5A–5F). For determining the amount of engrafted MSCs in the six groups, the real-time PCR for the male-specific *SRY* gene copy number was used to calculate the total number of the whole heart. According to the standard curve, there was higher percentage of engrafted MSCs in RIPoC + IPoC group, RIPoC group and IPoC group than that in IR group (2.24±0.35%, 2.28±0.42% and 2.22±0.44% vs. 1.44 ±0.26%, respectively, p<0.05)(Fig 5G). The MSCs retention in NAC+IR group(1.81 ±0.26%) was higher than that in IR group(p<0.05), but lower than that in RIPoC + IPoC group, RIPoC group and IPoC group(p<0.05).

### The enhancement of cardiac function

LVESD was significantly reduced in RIPoC+IPoC group, RIPoC group and IPoC group in comparison with that in IR group (4.66±0.29, 4.74±0.43 and 4.60±0.45 vs. 5.66 ± 0.21mm, p<0.01) at 3 weeks after MSC delivery. LVFS was higher in RIPoC+IPoC group, RIPoC group and IPoC group in comparison with that in IR group (43.5±4.28%, 42.4±4.9% and 43.5±4.6% vs. 32.0±1.9%,p<0.01) at 3 weeks after MSC delivery (Fig 5H). LVEDD showed no significant difference in any of the cell transplantation groups except that in sham group.

### The percentage of fibrosis

**Fig 6:**
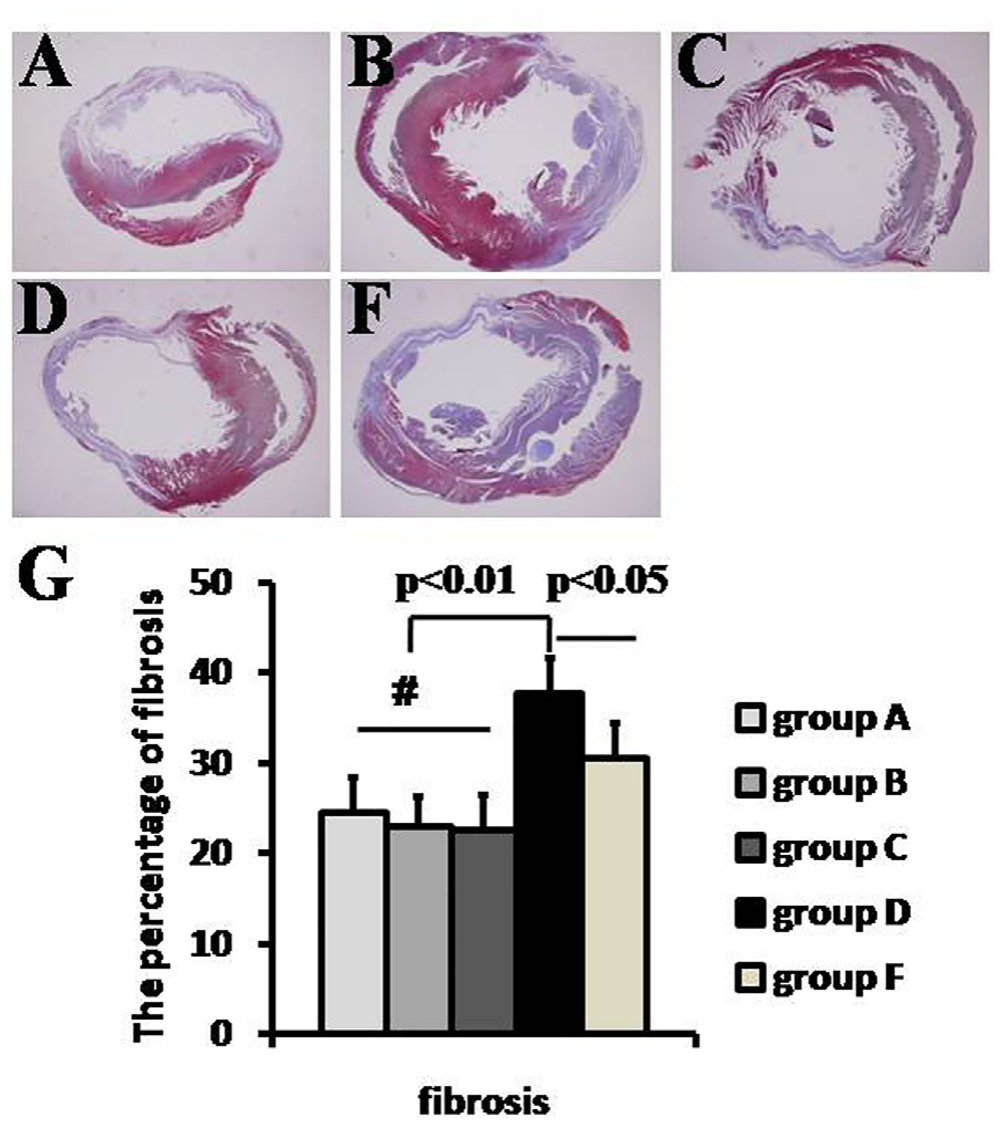
Cardiac fibrosis determination. Cardiac fibrosis (A-D, F) was compared among five groups at 3 weeks after mesenchymal stem cells(MSCs) delivery(n = 4). Cardiac fibrosis was less in RIPoC+IPoC, RIPoC and IPoC group than that in IR group(p<0.01). Group A: RIPoC+IPoC group, received the combined algorithms of RIPoC and IPoC; Group B: RIPoC group, which received 3 cycles of 30 seconds reperfusion and re-occlusion on the limb at the onset of reperfusion; Group C: IPoC group, received 3 cycles of 30 seconds reperfusion and re-occlusion on the LAD; Group D: IR group, received reperfusion without interruption after ischemia; Group F: NAC+IR group, ROS scavenger N-acetylcyseine was administered in IR group.

At 3 weeks after MSC delivery, histological analysis indicated that there was significantly less fibrosis of LV in RIPoC + IPoC group, RIPoC group and IPoC group than that in IR group (24.5±3.9%, 23.0±3.2% and 22.5±4.0% vs. 37.7±3.9%, p<0.05) (Fig 6A–6D, 6F). The percentage of fibrosis was less in IR+NCA group (30.5± 3.9%) than that in IR group(p<0.01), but higher than that in RIPoC + IPoC group, RIPoC group and IPoC group(p<0.05).

## Discussion

In this study, we showed that RIPoC attenuated the reperfusion injury and enhanced the retention of the intramyocardially delivered MSCs in rat IR model. This effect on MSCs retention rendered by RIPoC was consistent with the enhancement of cardiac function and the decrease of cardiac fibrosis. Meanwhile, the beneficial effect elicited by brief ischemia and reperfusion administered on the limb could be not added on by IPoC.

Allogeneic MSCs transplantation has emerged as a therapeutic modality in cardiovascular disease due to the inherent immunomodulatory properties [6]. The recent paradigm shift was seen in therapeutic use of MSCs through trophic mechanisms based on paracrine and endocrine mechanisms as opposed to their multi-lineage differentiation capacity [15]. Thus, the functional efficacy of transplanted cells is supposedly related to the amount in ischemic tissue. However, low cell engraftment and survival in myocardial milieu are still the main restraints in current experimental and clinical studies with all the available cells therapy [8].Although intramyocaridal MSCs transplantation is most efficacious compared with other delivery routes, the retention is still less than optimal [16]. Apart from cell evasion from the injected site [17,18], the hostile milieu in ischemic myocardium which ischaracteristic of ischemia, inflammation and oxidative stress was mainly accountable for severe loss. All kinds of methods have been resorted to enhance the therapeutics benefit, including cell pretreatment with hypoxia[19], tissue engineering and genetic manipulation. Due to their uncertainties and possible safety concerns, we explored to enhance the transplantation efficacy with the help of non-invasive clinical procedure.

Sustained improved survival obtained after reperfusion therapy in acute MI [20], but it also incurred oxidative stress and inflammatory reaction. It was documented that modifying the heart by gene depletion of PHD3, a pro-apoptotic protein, inhibits cardiomyocyte apoptosis and attenuates myocardial injury induced by ischemia-reperfusion [21]. RIPoC, several cycles of ischemia and reperfusion remote from heart at the timing of initiating reperfusion could also attain the cardioprotective effect without side effects. At the onset of reperfusion, opening of mitochondrial permeability transition pore(mPTP) in the inner mitochondrial membrane was favored by ROS generation and calcium overload. The opening of mPTP is the crucial event that may cause either apoptosis or necrosis of reperfusion myocardium [22–25]. RIPoC could reduce the deleterious effect and salvage the opening of mPTP [22,23].

To identify the extent of injury in different reperfusion modal**s**, multiple parameters were analyzed. Firstly, oxidative reaction and lipid peroxidation were compared, which indirectly suggested the reactive oxygen species(ROS) generation and calcium overload, respectively. ROS is important effector to ignite the cascade of oxidative stress, and it was also showed to inhibit cellular adhesion of the engrafted MSCs [26]. Calcium overload mainly results from cell membranes subject to lipid peroxidation. Secondly, as cellular ATP is produced in the mitochondria, mitochondrial dysfunction in cardiomyocytes and inflammatory cell infiltration around infarction area also existed. Energy depletion leads to left ventricular contractile insufficiency. The loss of ATP content and broken energy transfer system are well known markers for energy depletion[13]. Nextly, the activated level of Akt and Erk1/2 were calculated as two important proteins in the RISK pathway which played a pivotal role in the opening of mPTP. As conditioned hearts in previous study, Akt but not ERK1/2 was the predominant kinase in RIPoC-mediated cardioprotection[27]. Finally, the gene expression of SDF-1α and VEGF in the ischemic area was determined, which could contribute to stem cells retention and survival after cell delivery because they are characteristic of homing and angiogenesis, respectively. Moreover, the previous study indicated that VEGF could attenuate the reperfusion injury [28].

As RIPoC attenuated the harsh milieu deteriorated by reperfusion injury, we assumed RIPoC could enhance MSCs engraftment and increase survival rate in the ischemic zone. Enhancement of MSCs engraftment could exert more biological function through trophic mechanisms. By Brdu-labeled tracing method analysis and sex mismatch transplantation protocol, we assessed the cell retention in myocardium and identified more MSCs in RIPoC group than that in IR group. IPoC could not add the overlying effect on reducing reperfusion injury exerted by RIPoC. The seemingly two disparate conditioning sources may converge directly or indirectly on the cardiomyocyte mitochondria as a final effector [5]. It was also demonstrated that combination RIPoC with IPoC strategy did not render more MSCs retention or extra heart function recovery, as the combination of remote ischemic perconditioning and local ischemic postconditioning did not provide an additional reduction in infarct size in ST-elevation myocardial infarction patients [6].

To determine the effect of oxidative stress injury in myocardium on MSCs retention in the setting of IR, we quantified the intramyocardially delivered MSCs with the administration of ROS scavenger N-acetylcyseine (NAC). We did observe more retention after NAC preconditioning compared with that in IR group, but lower retention than that in RIPoC group. Moreover, the anti-oxidative stress reagent didn’t enhance the cardiac function after IR injury. It demonstrated that the effect of RIPoC on anti-oxidative stress in IR injury was important but not the only mechanism. In contrast to the vitro cellular hypoxic priming, which was characteristic of enhancing tolerance mechanisms and paracrine activity by preconditioning the MSCs [29], RIPoC enhanced MSCs therapeutic efficacy by modifying the tissue microenvironments. The shared objective of the two kinds of modality was to protect the engrafted MSCs and to enhance cells survival after transplantation, because it is generally thought that the engrafted cells exert on cardioprotective effect in the dose-dependable manner irrespective of their functional mechanism. However, cell transplantation followed by priming of local milieu had an advantage over cell preconditioning because cells could not be potentially undermined.

This study is of clinical significance because RIPoC could be used as an adjunctive therapy along with cell transplantation therapy while renewing coronary artery reperfusion of ischemic myocardium, such as in the clinical setting of cardiac surgery. The surgeon can concentrate on the overall procedure while anesthetist can manipulate a tourniquet on the upper or lower limb while restoring the blood reperfusion. It avoids the interruption of operation when IPoC is being administered during the clinical procedure.
